# Endolysosomal TRPMLs in Cancer

**DOI:** 10.3390/biom11010065

**Published:** 2021-01-06

**Authors:** Mengnan Xu, Xian-Ping Dong

**Affiliations:** Departments of Physiology and Biophysics, Dalhousie University, 5850 College Street, Halifax, NS B3H 4R2, Canada; mn863235@dal.ca

**Keywords:** endolysosome, ion channel, calcium, autophagy, cancer

## Abstract

Lysosomes, the degradative endpoints and sophisticated cellular signaling hubs, are emerging as intracellular Ca^2+^ stores that govern multiple cellular processes. Dys-homeostasis of lysosomal Ca^2+^ is intimately associated with a variety of human diseases including cancer. Recent studies have suggested that the Ca^2+^-permeable channels Transient Receptor Potential (TRP) Mucolipins (TRPMLs, TRPML1-3) integrate multiple processes of cell growth, division and metabolism. Dysregulation of TRPMLs activity has been implicated in cancer development. In this review, we provide a summary of the latest development of TRPMLs in cancer. The expression of TRPMLs in cancer, TRPMLs in cancer cell nutrient sensing, TRPMLs-mediated lysosomal exocytosis in cancer development, TRPMLs in TFEB-mediated gene transcription of cancer cells, TRPMLs in bacteria-related cancer development and TRPMLs-regulated antitumor immunity are discussed. We hope to guide readers toward a more in-depth discussion of the importance of lysosomal TRPMLs in cancer progression and other human diseases.

## 1. Introduction

**Figure 1:**
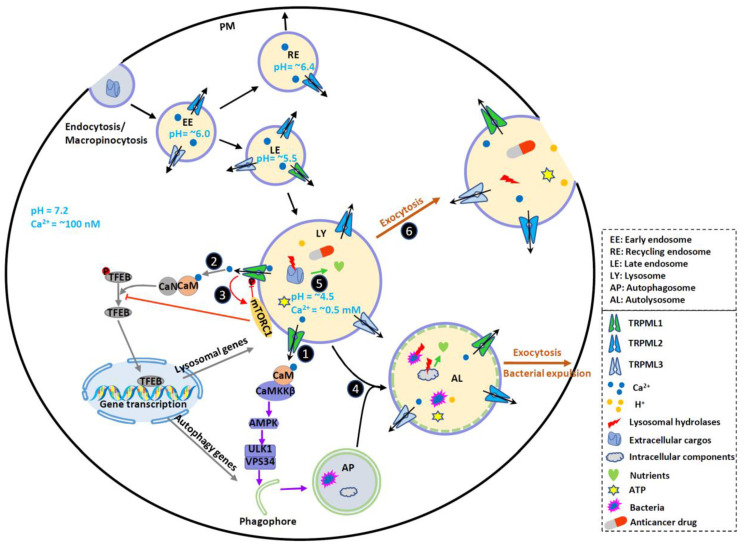
TRPMLs in endocytic, phagocytic and autophagic pathways and tumor progression. TRPMLs are predominately located on the endolysosomal pathway, and they have been involved in endocytic, phagocytic and autophagic pathways. In the tumor microenvironment, autophagy is activated to help cancer cells digest damaged or nonessential proteins and organelles, meeting the increased energy and nutrient demand of cancer cells. By releasing intraluminal Ca^2+^, TRPMLs have been implicated in intracellular Ca^2+^ signaling, endolysosome trafficking, and lysosomal functions, further regulating autophagy. First, TRPML1 activates calmodulin (CaM)/CaMKKβ/AMPK pathway to promote autophagosome formation. Second, TRPML1 activates CaM/CaN/TFEB pathway to continuously supply lysosome and autophagy proteins. Third, TRPML1 maintains mTORC1 activity to prevent cancer cell death and promote lysosome reformation. In normal fed conditions, mTORC1 phosphorylates TRPML1 at S571 and S576 to inhibit its activity. Starvation reduces mTORC1 activity and disinhibits TRPML1. This subsequently promotes mTORC1 activity, preventing cell death. Fourth, TRPML1 stimulates Apoptosis-linked gene-2 (ALG-2)-dependent lysosome centripetal movement to facilitate autophagosome-lysosome fusion. Fifth, TRPML1 increases lysosomal degradative functions, likely through controlling lysosomal pH. Sixth, TRPML1 increases Syt7-dependent lysosomal exocytosis, releasing hydrolases, ATP and H^+^ to extracellular spaces. These may change tumor microenvironment, promote ECM degradation, and facilitate tumor progression. By promoting lysosomal exocytosis, TRPML1 may also participate in drug resistance by releasing sequestrated anticancer drugs. Due to the functional redundancy between the TRPML proteins, TRPML2 and TRPML3 may also contribute to some of these events to regulate cancer development. Therefore, TRPMLs orchestrate all these cellular events to help cancer cells maintain high autophagic flux and adapt to the tumor microenvironment.

**Figure 2:**
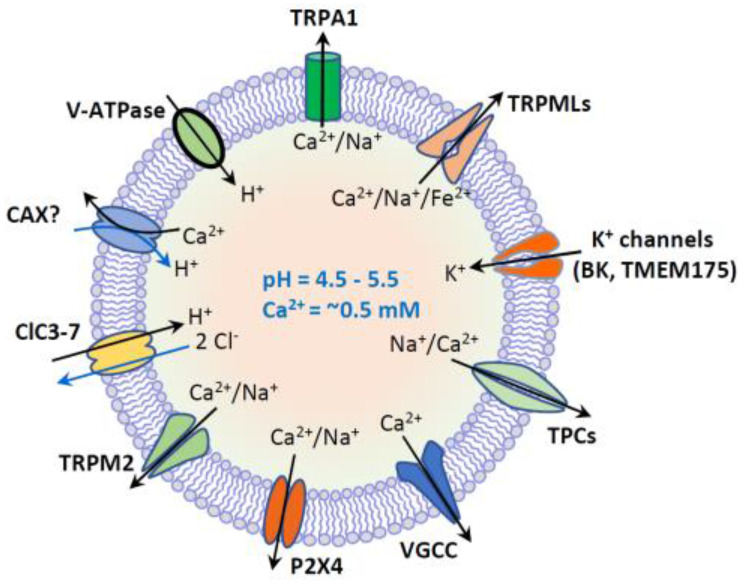
Lysosome ion homeostasis and ion channels. The lysosome has an acidic lumen that contains soluble hydrolytic enzymes. The activity of hydrolytic enzymes is controlled by intraluminal ion homeostasis that is established by multiple ion channels and transporters. These ion channels and transporters include H^+^-ATPase, nonselective cation channels (TRPML1-3, TRPM2, TRPA1 and P2 × 4), Na^+^ or Na^+^/Ca^2+^-selective two-pore channels (TPC1-3), voltage-gated Ca^2+^ channels (VGCC), K^+^-selective channels (BK and TMEM175), and 2Cl^−^/1H^+^ exchanger or Cl^−^channels (ClC3-7). Putative Ca^2+^/H^+^ exchanger (CAX) or Ca^2+^ transport protein mediates lysosomal uptake of Ca^2+^. Lysosomal Ca^2+^ (~0.5 mM) is important for membrane trafficking, and lysosomal pH (4.5–5.5) is essential for the activity of hydrolytic enzymes.

Lysosomes, the membrane-bound digestive cell organelles, are well-known for their catabolic function. They dynamically interact with autophagy and endocytosis pathways which transport intracellular components or extracellular cargos to lysosomes, respectively [1,2,3,4] (Figure 1). Both intracellular and extracellular cargos can be broken down into their constituent building blocks (e.g., amino acids and free fatty acids) by lysosomes through hydrolysis [5,6]. To date, around 60 hydrolytic enzymes, including proteases, lipases and nucleases have been found in lysosomes [7]. The activity of these hydrolytic enzymes is stimulated by the acidic lumen (pH 4.5–5.5) of lysosomes, which is established by the vacuolar H^+^-ATPase (V-ATPase), an ATP-driven proton pump [5,8,9] (Figure 2). Metabolites produced by hydrolysis are eventually recycled back to the cytoplasm or exported to the extracellular environment through exocytosis (Figure 1).

In addition to being the degradative endpoints, lysosomes are also the cellular signaling hubs that are involved in the regulation of cell growth, proliferation and differentiation [6,7,10,11]. For example, the lysosomal membrane hosts nutrient- and energy-sensing machineries in response to both internal stimuli and external changes of environment. In particular, both the master nutrient sensor mechanistic target of rapamycin complex 1 (mTORC1) [6,12,13,14,15,16] and the master energy sensor AMP-activated protein kinase (AMPK) [17,18,19,20] are associated with lysosomes and strictly control cell growth, proliferation and differentiation by sensing cellular metabolic status [17,21].

Lysosomal degradation and signaling require the establishment of the luminal ionic homeostasis [1] including Ca^2+^, Na^+^, K^+^, Cl^−^ and heavy trace metals such as Fe^2+^ and Zn^2+^. The lysosome is one of the main storage organelles of the second messenger Ca^2+^. The luminal Ca^2+^ concentration of lysosome is ~0.5 mM [22,23], which is approximately 5000-fold higher than that in cytosol (~100 nM) [24] (Figure 1). The Ca^2+^ gradient across the lysosomal membrane is important, as it enables a small fraction of lysosomal Ca^2+^ efflux to generate a marked signal, resulting in the activation of downstream signaling cascades [2,25,26,27]. Lysosomes express various Ca^2+^-permeable channels on their membranes to regulate both local and global intracellular Ca^2+^ signals. These Ca^2+^-permeable channels include TRP Mucolipins (TRPMLs, TRPML1-3), Two Pore Channels (TPCs, TPC1-2), TRP Melastatin 2 (TRPM2), TRP Ankyrin 1 (TRPA1), P2X4 purinoceptor, and Voltage-Gated Ca^2+^ Channel (VGCC) [1,23,28,29,30,31,32] (Figure 2).

## 2. Endolysosomal TRPMLs

**Figure 3:**
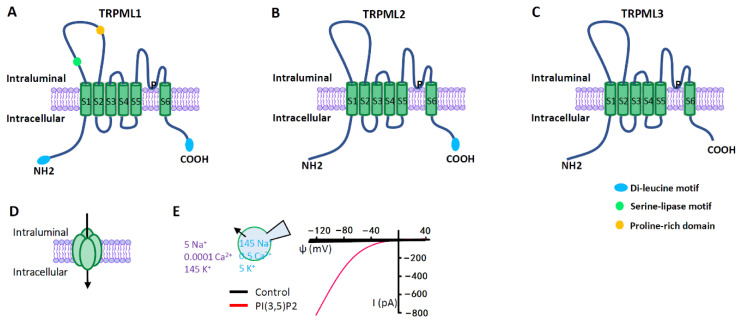
TRPML ion channels. (**A**–**C**) TRPML pore-forming subunit contains six transmembrane segments (S1–S6) and a putative pore region (P), with presumably cytosolic N- and C- termini. Distinct from other ^TRP^ channels, TRPMLs are characterized by a large extracellular (or intraluminal) loop between S1 and S2. There are dileucine motifs in TRPML1 and TRPML2 at their C- and/or N- termini to determine their intracellular endolysosomal localization. The endolysosomal localization of TRPML3 is determined by its heteromultimerization with other TRPMLs. (**D**) Functional TRPMLs are tetramers. (**E**) TRPMLs currents measured using lysosome-patch-clamp method. Physiological asymmetric solutions are used. The bath solution (cytosolic) contains (in mM) 145 K^+^, 5 Na^+^, 0.0001 Ca^2+^, and pH 7.2. Pipette solution (luminal) contains (in mM) 145 Na^+^, 5 K^+^, 0.5 Ca^2+^, and pH 4.6. TRPMLs are activated by cytosolic PI (3,5) P2 (1 µM), a lysosome-specific phosphoinositide. TRPMLs are inwardly (cation flowing from the lumen to the cytosol) rectifying channels permeable to cations. Given the topology of the TRPML proteins at the lysosomal membrane and the electrical properties of the lysosome, TRPML opening leads to Ca^2+^ and Na^+^ release from the lysosome to the cytosol.

TRPMLs are the most intensively studied lysosomal Ca^2+^ channels, which belong to the large family of TRP ion channels [33,34,35] (Figure 3). Compared with other channels of TRP family that are expressed on plasma membrane (PM), TRPMLs act predominantly in the endolysosomal system, regulating vesicles trafficking and function along endolysosomal pathways. All three members of TRPMLs can be activated by PI (3,5) P2, an endolysosome-specific phosphoinositide [36]. In mammals, TRPML1 is expressed in all tissues [37,38], and it is predominately localized on the late endosome (LE) and lysosome [35,39] where the low luminal pH facilitates its activation [39]. By releasing lysosomal Ca^2+^, TRPML1 regulates several membrane-trafficking processes, including lysosome to trans-Golgi–network (TGN) retrograde trafficking, autophagosome-lysosome fusion, and lysosomal exocytosis [1,40,41,42]. Mutations in TRPML1 causes a human autosomal recessive disease named mucolipidosis type IV (MLIV), a lysosomal storage disease (LSD) [37,43]. Impaired TRPML1 has also been associated with several other LSDs [44]. Compared with TRPML1, TRPML2 and TRPML3 are less understood. Both of them are more easily activated by higher pH [45,46,47]. Although TRPML2 is found in most organs, it is abundant in immune cells and tissues [48,49,50]. Subcellularly, TRPML2 is primarily expressed on the recycling endosomes (RE) and the early endosomes (EE) [46,50,51] where it regulates the recycling of specific proteins from RE/EE to the cell surface [51]. Emerging evidence suggests that TRPML2 is an osmo/mechanosensitive ion channel on endolysosomal membranes [52]. Currently, TRPML2 has not been linked to any human disorders. However, it may play an important role in the secretion of chemokine and cytokine by macrophages [46,50]. TRPML3 is predominantly localized in the endocytic and autophagic pathways, in line with its cellular function of regulating endocytosis and autophagy [53,54,55]. The expression of TRPML3 has been detected in skin melanocytes, hair cells of the inner ear, neonatal intestinal enterocytes, as well as cells in the thymus, kidney and lung [56,57]. The gain-of-function mutation A419P in TRPML3 causes the varitint-waddler phenotype in mice, characterized by hearing loss and circling behavior [47,58,59]. 

Given the crucial role of lysosomes in multiple biological functions and signals, dysregulation of lysosomal function may cause human diseases. Indeed, a growing number of studies have demonstrated that tumor angiogenesis and progression are associated with altered lysosomes [60,61,62,63]. Growing evidence also suggests that intracellular Ca^2+^ signals mediated by lysosomal Ca^2+^ channels may control the development of various cancers. Herein, we summarize the emerging role of lysosomes and TRPMLs in cancer development. We hope to guide the readers into a more in-depth discussion of the relationship between lysosomal Ca^2+^ signaling and cancer development, potentially directing the development of new therapeutics for cancer.

## 3. Alterations of Lysosomes in Cancer Cells

Lysosomes in cancer cells display some differences from normal cells. These changes are often related to cancer cell growth, proliferation, invasion and drug resistance [61]. Currently, more than 20 clinical trials are aiming at evaluating the efficacy of lysosome inhibitors (e.g., chloroquine and hydroxychloroquine) in the treatment of different cancers [61,64,65,66,67,68].

### 3.1. Lysosomal Biogenesis in Cancer

The high-level proliferation of cancer cells heavily depends on necessary nutrients and energy that can be supplied by increased catabolic activity. Because both exogenous macromolecules and endogenous components can be digested and recycled by lysosomes to fuel cell growth [61], cancer cells are particularly dependent on the effective lysosomal function. To meet the needs of higher metabolic rates, cancer cells often display increased lysosomal biogenesis [69,70] to promote cancer cell growth and proliferation and protect cancer cells from stress conditions by increasing degradation and nutrient recycling [61,70,71] (Figure 1). 

### 3.2. Lysosomal Hydrolase Activity in Cancer

Enhanced expression and activity of lysosomal hydrolases have been commonly found in many human tumors [72,73,74,75,76]. The increased hydrolase activity could promote tumor progression by providing more nutrient and energy. These hydrolases can also be secreted to extracellular spaces to regulate tumor microenvironment [74,75]. For example, cathepsins, the best studied lysosomal hydrolases, can be secreted via exocytosis of peripheral lysosomes, supporting cancer cell migration and invasion [62,74] by degrading the extracellular matrix (ECM) [61,62]. In line with this, lysosomes are often delocalized to the periphery of cancer cells [62].

### 3.3. Lysosomes in the Alteration of Local Cancer Microenvironment

In addition to releasing lysosomal hydrolases to the extracellular microenvironment, lysosomal exocytosis can also cause changes of tumor microenvironment by releasing intraluminal components. For example, lysosomal protons could acidify the tumor microenvironment, which enables cathepsins to degrade ECM more effectively [62,77] and favors tissue damage and tumor invasion and progression. Lysosomal ATP can also be released to the extracellular environment through lysosomal exocytosis, promoting invasion and metastasis of triple-negative breast cancer (TNBC) [78] (Figure 1). 

### 3.4. Changes of the Lysosomal Membrane Proteins in Cancer

Vacuolar H^+^-ATPase (V-ATPase) is an important lysosomal membrane protein that is essential for the lysosomal acidification. The overexpression of V-ATPase subunits has been reported in several tumor tissues and cancer cell lines [79,80,81,82,83,84,85,86]. Interestingly, V-ATPase has also been found in the PM of several invasive cancer cells [79,81,82,83,84]. This may promote the acidification of tumor microenvironment by the extrusion of proton, potentially contributing to tumor invasion [87].

The expression of lysosomal ion channels is also altered in certain types of cancer cells. For example, a recent study has demonstrated that the mRNA levels of two-pore channels (TPCs) are particularly high in bladder cancer, hepatocellular carcinoma and leukemia cells, where TPCs are important for cancer cell migration and the dissemination of tumor cells [88]. An increase in the expression of TRPMLs in different cancers has also been suggested. In TNBC cell lines the expression of TRPML1 is upregulated compared with nontumorigenic cells and nonmetastatic ER^+^/PR^+^ breast cancer cells [78]. TRPML1 levels are also significantly elevated in *HRAS*-positive tumors [89]. The elevated TRPML1 promotes cancer development by increasing cancer cell growth, proliferation, invasion and metastasis [78,89]. The details of TRPMLs in cancer development will be discussed later. 

### 3.5. Destabilization of Lysosomal Membranes in Cancer

The loss of lysosomal membrane integrity allows the release of luminal contents into the cytosol, so called lysosomal membrane permeabilization (LMP). It is a mechanism for the induction of cell death in specific circumstances. Cell death triggered by LMP is gaining increased interest as target for cancer therapy. Although the increased lysosomal cathepsins may facilitate cancer progression by increasing lysosomal functions and ECM degradation, they can also destabilize lysosomal membranes, subsequently being leaked to the cytosol to kill cancer cells [90,91,92]. Therefore, the role of cathepsins in cancer development may depend on the stage of cancer development. In addition, many cancers have altered sphingolipid metabolism, which negatively affects lysosomal membrane structure and causes LMP [93,94,95]. Several cationic amphiphilic drugs (CADs) have been found to preferentially induce the damage of lysosomes in cancer cells by interfering with sphingolipid metabolism [62]. Other factors such as increased size of lysosomes [69,96] may also cause LMP. 

### 3.6. Lysosomal-Dependent Autophagy Pathway in Cancer

Autophagy is a lysosomal-dependent nutrient recycling process which contributes to cell survival. In response to starvation and other stresses, cells often initiate this catabolic pathway to digest damaged proteins and organelles to recycle nutrients (Figure 1). Alterations of autophagy have been shown in cancer cells, particularly in cancers bearing *RAS* mutations [97]. The association between cancer and autophagy is complex. Both antitumorigenic effects and protumorigenic roles of autophagy have been suggested. Current data supports a dynamic role of autophagy in cancer, impeding early cancer development while facilitating advanced tumor progression and maintenance [64,98,99,100,101,102,103,104,105]. The dual role of autophagy in carcinogenesis could be attributed to its role in the biogenesis of protumorigenic elements. At initial stages, autophagy prevents tumorigenesis by removing protumorigenic elements [e.g., high concentration of reactive oxygen species (ROS) induced by mitochondrial dysfunction and oncogenic protein aggregates] [106], while in developed tumor autophagy may favor tumor growth by removing the detrimental elements (e.g., impaired proteins and organelles), enhancing cancer cell survival and resistance against nutrient deprivation, hypoxia and chemotherapy [107], suppressing antitumor immunity [108], and increasing survival of dormant cells [109]. Given that mTORC1 and AMPK are key nutrient and energy regulators playing important roles in cellular metabolism, energy homeostasis, cell growth and differentiation, dysregulation of each pathway may contribute to cancer development [17,21,110,111,112,113]. 

### 3.7. Lysosomes in Anticancer Drug Resistance

One of the challenges of treating cancer is that cancer cells have the ability to develop drug resistance, a leading cause of the failure of chemotherapeutic treatment. Lysosomes have been suggested to mediate drug resistance in cancer. On the one hand, hydrophobic weak base anticancer drugs can be sequestrated by lysosomes. This further increases lysosomal drug sequestration capacity by increasing lysosomal fusion [114] or the TFEB-mediated lysosomal biogenesis [115,116]. On the other hand, cancer cells can compartmentalize anticancer drugs away from the cellular targets through lysosomal exocytosis [117,118], causing insensitivity of the cancer cells to treatment [118] (Figure 1). 

Collectively, along with tumor progression lysosomes change their number, subcellular distribution, stability, expression of membrane proteins and enzyme activity, and tumor microenvironment, thereby adapting to and surviving the tumor microenvironment, proliferating, growing and metastasizing. Developing drugs targeting on different lysosome-related pathways could be potential treatment for cancers.

## 4. The Lysosomal Ca^2+^ Channel TRPMLs in Cancer

The dysregulation of lysosomal Ca^2+^ channels has been associated with a variety aspects of cancer development, including tumorigenesis, tumor growth and metastasis [88,119]. However, our understanding of the association of TRPMLs with cancer is still limited. In this section, we provide an overview on the roles of TRPMLs in cancer development. 

### 4.1. The Expression of TRPMLs in Cancer

Mutation of *RAS* oncogenes is a leading causes of cancer [120]. A recent study suggests that TRPML1 expression is significantly elevated in cancer cells bearing oncogenic *HRAS* mutations, and TRPML1 expression is inversely correlated with patient prognosis [89]. These tumor cells are vulnerable to both TRPML1 knockdown and TRPML1 inhibition. Mechanistically, TRPML1 plays an important role in maintaining oncogenic *HRAS* at the PM through regulating cholesterol homeostasis, promoting the growth of cancers [89]. In the meantime, another study suggested that TNBC cell lines express higher levels of TRPML1 compared with nontumorigenic cells and nonmetastatic ER^+^/PR^+^ breast cancer cells [78]. By using cell models and an animal model, it was suggested that elevated TRPML1 in TNBC promotes cell proliferation by activating mTORC1 and facilitates cell invasion and metastasis by facilitating lysosomal ATP exocytosis. Consistently, a later study suggested that TRPML1 expression is elevated in melanoma cells relative to melanocytes, and TRPML1 is preferentially required for the survival and proliferation of melanoma cells [121]. Distinct from TNBC, in melanoma TRPML1 negatively regulates mTORC1 signaling to sustain macropinocytosis and protein homeostasis [121]. These studies suggest although the enhanced level of TRPML1 promotes cancer progression, the underlying mechanisms could be different depending on the types of cancer.

The link between TRPMLs expression and the clinical characteristics of patients with cancer has also been reported. Yin et al. [122] found that the expression of TRPML1 increases along with the progression of human nonsmall-cell lung cancer (NSCLC). Similarly, by evaluating the expression levels of TRPML1 in tumor tissues from 82 pancreatic ductal adenocarcinoma (PDAC) patients, Hu et al. found that a higher TRPML1 expression level is associated with the poor clinical characteristics of these PDAC patients [123]. Compared with patients with low TRPML1 expression, patients with high TRPML1 expression have significantly lower overall survival. A role of TRPML1 in PDAC progression is further confirmed by using a cell model showing that the proliferation of PDAC cells is dramatically blocked by TRPML1 depletion, and by using a mouse model showing that TRPML1 is required for the formation and growth of PDAC tumors [123]. Collectively, these studies suggest that TRPML1 is often upregulated in cancer cells to promote tumorigenesis. However, this is not always the case. A recent study suggested that activation of TRPML1 by its agonist in glioblastoma (GBM) cell lines reduces cell viability accompanied by induced caspase-3-dependent apoptosis. This is rescued by either blocking TRPML1 dependent Ca^2+^ release or the silence of TRPML1 [124]. Consistently, a strong association between the reduction of TRPML1 mRNA expression and short survival in glioblastoma patients has also been revealed [124]. 

An increased TRPML2 expression in cancer cells was also reported recently. Morelli et al. found that elevated levels of both mRNA and protein of TRPML2 are associated with the increased pathological grade of malignant gliomas. Elevated TRPML2 contributes to survival and proliferation of glioma cell lines, while loss of TRPML2 induces apoptotic cell death of glioma cells [125]. However, Jung et al. demonstrated that TRPML2 gene is only elevated slightly in HN31 oral cancer cells and plays a minor role in cancer cell proliferation [89]. Almamun et al. further suggested that the downregulation of TRPML2 gene due to DNA methylation contributes to the development of acute lymphoblastic leukemia (ALL) [126], a cancer which occurs most commonly in children between the ages of two and five [127]. Therefore, the role of TRPML2 in cancer progression is also dependent on the types of cancers.

The role of TRPML3 in cancer development has not been well studied. In a recent study, Wu et al. integrated and analyzed several pancreatic cancer datasets, establishing a nine-gene prognostic signature that is able to classify patients with pancreatic cancer into high- and low-risk groups and predict the overall survival [128]. Among these nine genes, TRPML3 is identified as one of the protective genes, as its expression is significantly downregulated in pancreatic adenocarcinoma tissues comparing with nontumor tissues [128]. Consistent with this finding, data from The Cancer Genome Atlas (TCGA) database revealed that a variety of cancers have downregulated TRPML3 [128]. Inversely, TRPML3 is reportedly upregulated in squamous cell carcinoma and hepatocellular carcinoma [129]. Thus, further investigation is required to elucidate the role of TRPML3 in cancer development.

Although numerous studies have revealed an alteration of TRPMLs expression in a variety of cancers, our understanding of how TRPMLs regulate cancer progression is very limited. Next, we will summarize our current knowledge of the mechanisms underlying TRPMLs regulating cancer development from different aspects. We hope this will guide the development of treatment for different tumors by targeting TRPMLs. 

### 4.2. TRPMLs in Cancer Cell Nutrient Sensing 

The high level of proliferation is one of characteristics of cancer cells, as their normal cell-cycle control is often disrupted. The fast proliferation of cancer cells heavily depends on the supplies of cellular nutrient and energy. In addition, other processes of cancer development such as migration and invasion also need energy supplies. Thus, it is important for cancer cells to acquire sufficient nutrients and energy during their development. However, the nutrient and energy supplies cannot always meet the demand of rapid expansion of solid tumors. Therefore, it is essential for tumor cells to develop alternative mechanisms of nutrient acquisition and improve survival. Autophagy is the nutrient recycling process that contribute to cell survival (Figure 1). In response to starvation and other stresses, autophagy is often initiated to digest damaged proteins and organelles to recycle nutrient and energy, although prolonged autophagy might cause cell death in some contexts. Autophagy level is particularly elevated in *RAS*-driven cancers [97,130]. Interestingly, TRPMLs are important players in autophagy. TRPML1 has been suggested to promote autophagy by facilitating multiple steps of autophagy, including autophagy induction [131], autophagosome-lysosome fusion [1,132], autolysosome degradation [133,134,135], and autophagic lysosome reformation (ALR) [27,133]. Inhibition of TRPML1 decreases autophagy activity and reduces proliferation, migration and invasion of NSCLC cells [122]. Recent studies have demonstrated that TRPML3 also plays an important role in regulating autophagy [53,54,55]. However, whether TRPML3 is involved in cancer development through regulating autophagy remains unknown.

mTORC1 is the well-known nutrient sensor and autophagy regulator that controls the balance between anabolism (e.g., the production of proteins, lipids, and nucleotides) and catabolism (e.g., autophagy), thus governs cell growth and proliferation [16]. The activation of mTORC1 is highly regulated by lysosomes since mTORC1 can only be activated when it is recruited to the surface of lysosomes [12,136]. As an important lysosomal cation channel, TRPML1 has been shown to regulate mTORC1 activity. The involvement of TRPML1 in mTORC1 signaling was first revealed in Drosophila [137]. Recent studies [27,138] further demonstrated that TRPML1 is required for sustained activity of mTORC1 under nutrient stress but not normal conditions [27]. Given that cancer cells constantly face stressful conditions, such as nutrient starvation and enhanced oxidative stress, the TRPML1-mTORC1 signaling pathway could play a crucial in cancer cell survival (Figure 1). Indeed, deletion of TRPML1 causes reduced mTORC1 activity, leading to decreased cell proliferation [78]. Consistently, in vivo model shows that TRPML1 knockdown leads to a smaller tumor and slower growth rate as compared to the control. This suggests TRPML1-mediated mTORC1 signaling plays a crucial role in tumor growth. The beneficial role of TRPML1-mTORC1 in cancer development may depend on cancer types because increased mTORC1 activity in some cancer cells could lead to higher proteotoxic stress, suppressing cancer development. For example, increased TRPML1 expression in melanoma cells elevates proliferation of melanoma cells by suppressing mTORC1 activity [121]. This increased proliferation is partially caused by improved proteostasis due to decreased mTORC1 activity [121]. Thus, although TRPML1-regulated mTORC1 signaling is important for cancer development, distinct mechanisms may exist in different types of cancer cells. 

In addition to autophagy, macropinocytosis also plays an important role in promoting cancer cell proliferation through acquiring nutrients from the extracellular environment [139,140,141] (Figure 1). During amino acid starvation, the engulfed extracellular proteins by macropinocytosis undergo catabolism, supplying cells with exogenous amino acids and supporting the proliferation [142]. Importantly, it has been shown PIKfyve and its effector TRPML1 contribute to the shrinkage of micropinocytosis and nutrient export from lysosomes [142]. These nutrients reactivate mTORC1 [140], which could trigger anabolism and promote cell proliferation. Moreover, an increased dependence of macropinocytosis-regulated nutrient recovery has been shown in high metabolic *RAS*-mutant cells during starvation [139]. This is consistent with the fact that cancer cells bearing *RAS* mutations are particularly vulnerable to TRPML1 inhibition [89].

Recently, Scotto Rosato et al. suggested that TRPML1 induces autophagosome biogenesis through activating the calcium-dependent kinase CaMKKβ and AMPK, which increase the activation of ULK1 and VPS34 autophagic protein complexes [131]. This finding suggests that TRPML1 may also affect cancer development by regulating AMPK pathway. Considering that TRPML1-mTORC1 [27] downregulates autophagy through inhibiting ULK1 and VPS34 complex [143,144], and that TRPML1-AMPK upregulates autophagy through activating ULK1 and VPS34 complex [131,144], TRPML1 may coordinate both mTORC1 and AMPK signaling pathways to regulate cancer development at different stages.

### 4.3. TRPMLs-Mediated Exocytosis in Cancer

Ca^2+^- and synaptotagmin 7 (Syt7)-dependent lysosomal exocytosis [145,146] has been suggested to play a crucial role in tumor progression and chemoresistance [147] (Figure 1). Inhibition of the lysosomal exocytosis suppresses the invasiveness and chemoresistance of aggressive sarcoma cells, while increased lysosomal exocytosis promotes the invasiveness and drug-resistance [147]. Given that TRPML1 is a key regulator of lysosomal exocytosis [42,148,149], the role of TRPML1 mediated lysosomal exocytosis in the context of cancer development has been further explored. Jung et al. suggest that the cholesterol recycling by TRPML1-mediated lysosomal exocytosis contributes to the proliferation of oncogenic *HRAS*-driven cancer cells [89]. Knockdown or inhibition TRPML1 suppresses the movement of cholesterol from endolysosomal vesicles to the PM [89]. Consequently, the PM cholesterol levels reduces, attenuating cell proliferation [89]. In line with this, our recent study suggested that lysosomes contain high levels of ATP that can be released to the extracellular spaces by TRPML1-mediated lysosomal exocytosis, promoting TNBC cell invasion and metastasis [78]. In addition, a recent study [150] suggested that Tetrabromobisphenol A (TBBPA), one of the most important brominated flame retardants (BFRs) [151], significantly promotes the migration and invasion of hepatocellular carcinoma cell line-HepG2 through TRPML1-dependent lysosomal exocytosis [150].

### 4.4. The Potential Role of TRPMLs in TFEB-Mediated Gene Transcription in Cancer

TFEB is a master transcriptional regulator of autophagic function, lysosomal biogenesis and metabolism [152,153]. TFEB is tightly regulated by two proteins associated with lysosomes, mTORC1 and TRPML1. In the presence of nutrients, mTORC1 phosphorylates and inhibits TFEB [152,154,155]; mTORC1 also inhibits TRPML1 through phosphorylation [27,156] (Figure 1). In the absence of nutrients mTORC1 inactivation stimulates TRPML1 and its downstream phosphatase calcineurin (CaN). Activated CaN dephosphorylates TFEB, causing nuclear translocation of TFEB and subsequent transcription of lysosomal and autophagic genes [152,157,158] (Figure 1). Interestingly, TRPML1 is also a downstream transcriptional target of TFEB [159]. Thus, TRPML1 and TFEB form a positive feedback loop to promote autophagy and lysosomal biogenesis upon starvation. 

In a recent study, the TFEB-dependent increase of lysosomal biogenesis and function was observed in PDAC [70]. Several other studies have also demonstrated that TFEB is associated with the growth [160], proliferation [161], migration [162,163] and metastases [161,163,164] of cancer cells. However, whether TRPML1 is involved in the TFEB-dependent cancer development remains unclear. Based on the fact that both lysosomal function and autophagy are involved in cancer development, it is conceivable that TRPML1 may play an important role in TFEB-dependent pathway in cancer cells. Intriguingly, in addition to constitutive activation of TFEB, PDAC cell lines also show intact mTORC1 signaling [70]. This raises a question how TRPML1 coordinates mTORC1 and TFEB to promote cancer progression if mTORC1 inhibits TFEB in cancer cells.

### 4.5. TRPMLs in Bacteria-Related Cancer Development 

A variety of factors, including bacterial and viral infection, can cause chronic inflammation. During an infection, immune cells are activated, leading to the production of type I interferons (IFNs), proinflammatory cytokines and chemokines and acute inflammation. This further prevents the spread of infection and promotes the clearance of pathogens. However, persistent infections can induce chronic inflammation, which increases the risk of cancer [165]. For example, chronic gastritis caused by the infection of *Helicobacter pylori* (*H. pylori*)*,* a common human bacterium identified to invade gastric epithelial cells, could lead to gastric cancer [166,167], while bladder infected by *E. coli* is associated with bladder carcinogenesis [168,169]. Increasing evidence suggests that TRPMLs may be implicated in bacteria-related cancers by regulating endolysosomal membrane trafficking and autophagy. *H. pylori* secretes the key virulence factor vacuolating cytotoxin (VacA) to create a protective intracellular niche for H. pylori by impairing host endolysosomal trafficking and autophagy [64,170,171]. *H. pylori* within this intracellular niche is also protected from antibiotic treatment, which leads to infection recrudescence after therapy [171]. However, the molecular mechanisms underlying VacA effects remain largely unknown. Emerging evidence suggests that VacA inhibits TRPML1 to disrupt endolysosomal trafficking and autophagy, thereby creating a protective intracellular niche for *H. pylori*. TRPML1 agonists reverse the toxic effects of VacA on endolysosomal trafficking and autophagy, promoting the clearance of intracellular bacteria [171]. Given that TRPML3 is also involved in endolysosomal trafficking and autophagy, a role of TRPML3 in *H. pylori* clearance was suggested recently. Hu et al. found that after infection *H. pylori* are sequestered and survived in autophagosomes with impaired lysosomal acidification. Vitamin D3 treatment upregulates TRPML3 expression, causing increased lysosomal Ca^2+^ release and improved lysosomal acidification [172]. This enables the host cells to eliminated *H. pylori* through the autolysosomal pathway (Figure 1). 

Urinary tract infections (UTIs) have been suggested to contribute to an increased risk for developing bladder cancer [173,174]. The vast majority of UTIs are caused by Uropathogenic strains of *E. coli* (UPECs), a heterogeneous group of pathogens [175]. Interestingly, Miao et al. recently showed that TRPML3 plays an important role in expelling UPECs from bladder epithelial cells (BECs) through exosome release pathway [176,177] (Figure 1). During the infection, UPECs are targeted by autophagy and trapped in autophagosomes, which further fuse with multivesicular bodies (MVBs), leading to amphisome formation. In the amphisome, the inner autophagosomal membrane around the UPECs keeps intact and fuses with intraluminal vesicles (ILVs). The amphisome further fuses with lysosomes. However, these UPECs are not degraded in the lysosome due to their ability to neutralize lysosomal pH. In the meantime, the neutralized pH activates TRPML3 to release lysosomal Ca^2+^, resulting in the fusion of lysosome with the PM and the clearance of UPECs. Altogether, these studies suggest that TRPML1 and TRPML3 may represent therapeutic targets for *H. pylori* and UPEC infections and gastric and bladder cancers.

### 4.6. TRPMLs-Regulated Antitumor Immunity

Efficient function of the immune system is crucial to prevent cancer development because of its important role in immune surveillance of cancer and cancer suppression [178,179]. Growing evidence has suggested that TRPML1 plays an important role in a variety aspects of immune responses. For examples, Thompson et al. suggested TRPML1 is required for the transport of the Major Histocompatibility Complex II to the plasma membrane of macrophages [180]; Bretou et al. [181] reported that bacterial sensing by dendritic cells (DCs) activates TRPML1-TFEB to promote DCs migration to lymph nodes for antigen presentation to T cells.

Macrophages infiltrating tumor tissues or populated in the microenvironment of solid tumors are defined as tumor-associated macrophages (TAMs). As a critical component of tumor microenvironment, TAMs play multi-functional roles in tumor progression, including cancer growth, immune regulation, angiogenesis, metastasis, and chemoresistance [182,183,184,185]. Macrophages display functionally plasticity to accommodate different physiological conditions. Activated macrophages are often classified into classically-activated M1 macrophages and alternatively-activated M2 macrophages. In general, M1 macrophages foster inflammation response against invading pathogens and tumor cells, whereas M2 macrophages exert an immune suppressive phenotype, favoring tissue repair and tumor progression. A recent study reported that the TRPML1-TFEB pathway regulates antitumor immune response through resetting TAMs toward tumor-killing M1 phenotype [186]. 

TRPML2 is also involved in antitumor immunity. Tumor cell-derived microparticles (T-MPs), which contain tumor antigen profiles, can be recognized and taken up by DCs, leading to the presentation of multiple antigens to T cells for antitumor immunity [187]. This process is likely regulated by TRPML2 activity [188]. Mechanistically, T-MPs are endocytosed by DCs and then transported to lysosomes where T-MPs increase lysosomal pH via NOX2-catalyzed ROS production. The enhanced lysosomal pH plays an important role in the formation of MHC class I-tumor antigen peptide complexes. In parallel, T-MPs-induced ROS activates TRPML2, causing lysosomal Ca^2+^ release and subsequent TFEB activation. Activated TFEB directly binds to CD80 and CD86 promoters, promoting gene expression. This enables DCs efficiently present tumor antigen obtained from T-MPs to CD8+ T cells.

## 5. Conclusions

Since the discovery of the connection between cancer and the lysosome, targeting lysosomes has become an increasingly attractive cancer therapeutic strategy. However, because lysosomes are the degradative centers and sophisticated cellular signaling hubs, they play essential rules in both normal and cancer cells. Thus, it is crucial to minimize the potential side effects when developing anticancer drugs targeting lysosomes. 

Given that the lysosomal Ca^2+^ channel TRPML1 is specifically activated in the tumor microenvironment [27] and it plays important roles in the development of many cancers, instead of targeting the lysosome, inhibiting TRPML1 channel could be a more feasible approach to treat some cancers, especially for certain cancers with significant increase in TRPML1 expression, such as those bearing *HRAS* mutations [89] and TNBC [78]. To date, several membrane-permeable and structurally-unrelated ML1 antagonists (ML-SIs) [42,189] have been discovered. By improving their specificity, potency and efficacy using medicinal chemistry, we expect that some ML-SIs-based drugs will be developed. 

Nevertheless, our understanding on the role of TRPMLs in cancers is still limited. More detailed mechanisms await further investigation. In the coming years, the discovery of more potent and specific compounds targeting each TRPML isoform will surely facilitate the development of improved therapeutics for some cancers.

## Abbreviations

ALG-2	apoptosis-linked gene-2
ALL	acute lymphoblastic leukemia
ALR	autophagic lysosome reformation
AMPK	AMP-activated protein kinase
BECs	bladder epithelial cells
BFRs	brominated flame retardants
CADs	cationic amphiphilic drugs
CaM	calmodulin
CaN	calcineurin
DCs	dendritic cells
ECM	extracellular matrix
GBM	glioblastoma
H. pylori	Helicobacter pylori
IFNs	interferons
ILVs	intraluminal vesicles
LMP	lysosomal membrane permeabilization
LSD	lysosomal storage disease
MLIV	mucolipidosis type IV
mTORC1	mechanistic target of rapamycin complex 1
MVBs	multivesicular bodies
NSCLC	nonsmall-cell lung cancer
PDAC	pancreatic ductal adenocarcinoma
PM	plasma membrane
ROS	reactive oxygen species
Syt7	synaptotagmin 7
TAMs	tumor-associated macrophages
TBBPA	tetrabromobisphenol A
TFEB	transcription factor EB
TGN	trans-Golgi-network
T-MPs	tumor cell-derived microparticles
TNBC	Triple negative breast cancer
TPCs	two pore channels
TRP	transient receptor potential
TRPA1	TRP ankyrin 1
TRPM2	TRP melastatin 2
TRPMLs	TRP mucolipins
UPECs	uropathogenic Escherichia coli
UTIs	urinary tract infections
VacA	vacuolating cytotoxin A
V-ATPase	vacuolar H^+^-ATPase
VGCC	voltage-gated Ca^2+^ channel
